# Efficacy and safety of the paul glaucoma implant in the treatment of refractory primary congenital glaucoma

**DOI:** 10.1007/s10384-024-01076-0

**Published:** 2024-06-27

**Authors:** Murat Karapapak, Ali Olgun

**Affiliations:** 1 Department of Ophthalmology, University of Health Sciences, Basaksehir Cam and Sakura City Hospital, Istanbul, Turkey; 2 Department of Ophthalmology, West Eye Hospital, Erbil, Iraq

**Keywords:** PAUL glaucoma implant, Primary congenital glaucoma, Refractory glaucoma, Intraocular pressure, Surgical outcomes

## Abstract

**Purpose:**

To evaluate the safety and efficacy of the PAUL Glaucoma Implant (PGI) for managing refractory primary congenital glaucoma (PCG) over a one-year period.

**Study Design:**

Retrospective.

**Methods:**

A study was conducted using the medical records of thirty eyes of 17 patients who underwent PGI surgery for the treatment of refractory PCG. Primary outcome measures included failure criteria such as intraocular pressure (IOP) > 21 mm Hg, < 20% IOP reduction, necessity for further glaucoma intervention, implant removal, or loss of vision. Secondary outcomes focused on mean IOP, average number of glaucoma medications, best corrected visual acuity (logMAR), and incidence of complications.

**Results:**

The mean preoperative IOP of 38.8 ± 9.2 mmHg significantly decreased to 16.1 ± 3.3 mmHg at 12 months postoperatively (*p* < 0.001). The average number of glaucoma medications reduced from 3.6 ± 0.5 preoperatively to 0.9 ± 1.2 at 12 months post-op. Visual acuity remained stable in 24 eyes, decreased in 4, and increased in 2. Early postoperative complications occurred in 13.3% of patients, but no late complications were reported. The cumulative success rate was 86.6%.

**Conclusion:**

The PGI appears to be a safe and effective option for managing refractory primary congenital glaucoma, demonstrating significant IOP reduction and decreased dependence on glaucoma medications over a one-year period, with a high success rate and manageable complication profile.

## Introduction

Primary congenital glaucoma (PCG) stands as a rare pathological entity, stemming from aberrations in the developmental processes governing the trabecular meshwork, Schlemm’s canal, and the anterior chamber angle [1]. The escalated intraocular pressure (IOP) induces biomechanical stress on the pliable structures of the developing infant eye, resulting in a distinctive clinical feature known as buphthalmos.The corneal haziness, coupled with potential refraction errors, imposes significant impediments on the visual development of children affected by PCG.The optic nerve becomes susceptible to irreversible damage under the constant pressure exerted by the accumulated aqueous humor.

PCG poses a significant risk of irreversible visual impairment, often proving refractory to conventional medical interventions. The primary modality for managing PCG remains surgical intervention. The surgical approach involves the manipulation of the compromised drainage system, often through the opening or bypassing of the malformed Schlemm’s canal [2]. While procedures like goniotomy and trabeculotomy demonstrate favorable early success rates, a substantial proportion, approximately 20%, encounter failure over time, necessitating additional surgical interventions for sustained IOP control in affected children [2, 3]. Trabeculectomy with or without adjunctive antimetabolites has emerged as a promising avenue [4, 5]. Notably, trabeculectomy with mitomycin C has garnered attention due to reported success rates ranging from 52 to 95% [6, 7]. Despite the encouraging outcomes, the incorporation of antimetabolites introduces its own set of challenges. Long-term complications, such as bleb failure, bleb leak, and bleb-related endophthalmitis, have been recognized as drawbacks associated with the enhanced success rates achieved through these interventions [8].

Nonpenetrating external trabeculectomy presents an alternative approach, aiming to mitigate complications related to conventional trabeculectomy [9]. Moreover, glaucoma drainage devices have been considered, representing a viable option in cases where traditional filtration surgery proves insufficient. In a study of a single Ahmed glaucoma valve (AGV) implant in patients with PCG, the cumulative probability of success decreased to 56.3% at 5 years [10]. Cyclodestructive procedures stand as another facet in the armamentarium against PCG, targeting the ciliary body to reduce aqueous humor production [11]. However, the use of cyclodestructive measures necessitates careful consideration of potential side effects and long-term consequences.

The PAUL glaucoma implant (PGI) is a GDD designed to address and minimise post-operative complications associated with existing shunts. This device has demonstrated successful outcomes in the management of adult glaucoma [12, 13]. Notably, its tube dimensions distinguish it from established counterparts like the Molteno, Ahmed, and Baerveldt tubes. With an external diameter of 0.467 mm and an internal diameter of 0.127 mm, the PGI tube is designed to occupy less space in the anterior chamber angle, thereby reducing interference with surrounding structures. Despite the smaller tube dimensions, the PGI boasts a substantial surface area end plate for aqueous absorption, measuring 342 mm^2^. The configuration of the PGI plate is another notable feature, measuring 44.9 mm in length, and 23 mm in width. This unique design ensures that a lesser portion of the device lies beneath the recti muscles, potentially minimizing post-operative complications related to device positioning. The advantageous combination of smaller tube diameters and a large plate surface area addresses concerns related to hypotony while maintaining effective IOP control [13].

While the PGI has demonstrated favorable outcomes in adult cohorts, its application in the treatment of refractory PCG represents a novel frontier. This article presents 1-year data on the safety and efficacy of PGI in the context of refractory PCG.

## Methods

This study was reviewed and approved by the West Eye Hospital and conducted adhering to the ethical standards set forth in the Declaration of Helsinki. A total of thirty eyes of 17 patients who underwent PGI from June 2019 to November 2022 for refractory PCG were retrospectively analyzed. Inclusion criteria were an uncontrolled IOP (> 21 mmHg) despite the application of three topical antiglaucomatous drugs and a history of at least one prior glaucoma surgery. Patients with less than 12 months of postoperative follow-up and those lacking preoperative light perception were excluded. The primary outcome measures of the study were defined as failure when characterized by criteria such as an IOP greater than 21 mm Hg, reduction in IOP less than 20%, the necessity for implant removal, requirement for further glaucoma intervention, or loss of vision. Secondary outcome measures included the mean IOP, average number of glaucoma medications used, logMAR visual acuity, and incidence of complications. Patients underwent a thorough ocular examination, which included best-corrected visual acuity (BCVA), IOP measurements using Goldmann applanation tonometry, slit lamp examination of anterior and posterior segments, and gonioscopy. These assessments were performed preoperatively and at intervals of 1 week, 1 month, 3 months, 6 months, and 12 months postoperatively. BCVA was assessed using Teller acuity cards for patients for whom the Snellen chart could not be utilized. In cases where Goldman applanation tonometry could not be applied to patients enrolled in the study, we opted to measure intraocular pressure using Perkins tonometry under general anesthesia. For some patients, additional examinations were conducted as deemed necessary by the attending physician. In instances of elevated IOP, appropriate medical treatment was administered. If required, additional surgical procedures were undertaken.

### Surgical technique

PGI (Advanced Ophthalmic Innovations) was placed under general anesthesia in all patients. The PGI was predominantly placed in the superior temporal quadrant, while some patients received placement in the superior nasal quadrant. Following dissection of the conjunctiva and Tenon’s capsule, the PGI endplate was positioned beneath the rectus muscles. Subsequently, the endplate was securely sutured to the sclera using 6/0 vicryl, approximately 10 mm posterior to the limbus. The tube, adjusted to a few millimeters in the anterior chamber with the bevel oriented upward, underwent anterior chamber entry via a 26-gauge needle, parallel to the plane of the iris. The 6/0 monofilament prolene suture was carefully maneuvered from the plate using forceps, ensuring that approximately 1 mm remained within the anterior chamber. To aid in the advancement of the prolene suture into the tube, viscoelastic material was injected into the tube. This facilitated the smooth movement of the prolene suture within the tube, ensuring proper placement and function. The tube was then inserted into the anterior chamber through the needle pathway, positioned just above the iris, and carefully away from the corneal endothelium. The tube was covered along its length with pericardium. Tenon duplication was performed, and the pericardium was sutured to the sclera using 8/0 vicryl. The conjunctiva was then closed with 8/0 vicryl to complete the surgical procedure. All patients undergoing PGI surgery received standard postoperative care, including routine topical treatment with prednisolone and moxifloxacin for an extended duration of 8 weeks. At the 1st month postoperative follow-up, the previously placed ripcord sutures embedded under the conjunctiva were carefully removed under general anaesthesia.

### Primary and secondary outcomes

The primary outcome measure was defined as treatment failure, characterized by any of the following criteria: IOP values falling outside the target range of 5–21 mmHg, or less than a 20% reduction from baseline sustained over two consecutive visits after a 3-month period. Additionally, failure included the necessity for de novo glaucoma surgery, such as cyclodestructive procedures or additional tube shunt placement, removal of the implant, severe vision loss attributable to the surgery (e.g., endophthalmitis, suprachoroidal hemorrhage leading to vision loss), or progression to no light perception for any reason. The time to failure was operationally defined as the duration from the initial surgical intervention for glaucoma to the occurrence of reoperation for glaucoma, loss of light perception vision, or the first of two consecutive study visits, following a 3-month period, during which the patient exhibited either persistent hypotony (IOP ≤ 5 mm Hg) or inadequately reduced IOP (IOP > 21 mm Hg or not reduced by 20% below baseline).

The secondary outcome measures encompassed the evaluation of complications arising from the surgical intervention. Early complications were defined as manifesting at the 3-month follow-up visit, while late complications were identified subsequent to the 3-month follow-up. Procedures such as Ripcord suture removal, needling, and injection of viscoelastic gel into the anterior chamber were excluded from the definition of reoperation but were subject to evaluation. Additional outcome measures included the quantification of medications required throughout the follow-up period.

### Statistical analysis

Statistical analyses were performed with IBM SPSS Statistics (Version 28) software. Statistics related to continuous variables are reported as mean ± standard deviation and median (min-max) values, and descriptive statistics related to categorical variables are reported as numbers and percentages. Friedman’s test was utilised for more than two repeated measurements. Wilcoxon signed ranks test was used for preoperative and postoperative pairwise comparisons and Bonferroni correction was applied. Kaplan-Meier survival analysis method was used to determine the success rate. In the analyses, the significance level was 95% and the results were interpreted as statistically significant for a p value less than 0.05.

## Results

The study comprised thirty eyes of 17 patients who underwent PGI surgery for refractory PCG. Table 1 presents the demographic and clinical characteristics of these patients. At the time of surgery, the age of the patients ranged from 1 to 16 years, with a mean age of 7.7 ± 4.4 years. Among the patients, 52.9% were boys, and 47.1% were girls, and all were phakic. The mean axial length of the eyes was 25.1 ± 1.7 mm, the mean corneal diameter measured 13.4 ± 0.7 mm, and the mean cup-to-disc ratio was 0.9 ± 0.1. When previous glaucoma operations were analysed, 7 (23.3%) of the eyes had only trabeculectomy, 2 had only trabeculotomy and 2 had only micropulse transscleral diode laser cyclophotocoagulation (MP-TDLC). 19 eyes had a history of multiple operations. Among these eyes, 2 eyes had trabeculectomy, AGV, goniotomy and MP-TDLC, 3 eyes had trabeculectomy, goniotomy and MP-TDLC, 12 eyes had trabeculectomy and MP-TDLC and 2 patients had trabeculotomy and MP-TDLC.

**Table 1:** Demographics and clinical characteristics of participants

		Avg ± SD	Median(Min-Max)
Surgical age (Years)		7.7 ± 4.4	8.0 (1–16)
Sex	Male	9 (52.9%)	
	Female	8 (47.1%)	
Lens Status	Phakic	30 (100%)	
	Pseudophakic	0 (0%)	
	Aphakic	0 (0%)	
Axial Length (mm)		25.1 ± 1.7	24.8 (22.3–29.4)
Corneal diameter (mm)		13.4 ± 0.7	13.2 (12.5–14.8)
Cup-to disc ratio		0.9 ± 0.1	0.9 (0.6-1.0)
Preoperative BCVA (LogMAR)		0.87 ± 0.46	1.00 (0.0-1.3)
IOP (Preoperative)		38.8 ± 9.2	41.0 (24.0–55.0)
Number of anti-glaucoma medications (Preoperative)		3.6 ± 0.5	4.0 (3.0–4.0)

Continuous variables are presented as the mean ± standard deviation / median (min-max). Categorical variables are presented as number (%)

*AVG* average, *BCVA* best corrected visual acuity, *IOP* intraocular pressure, *SD* standard deviation

**Fig. 1:**
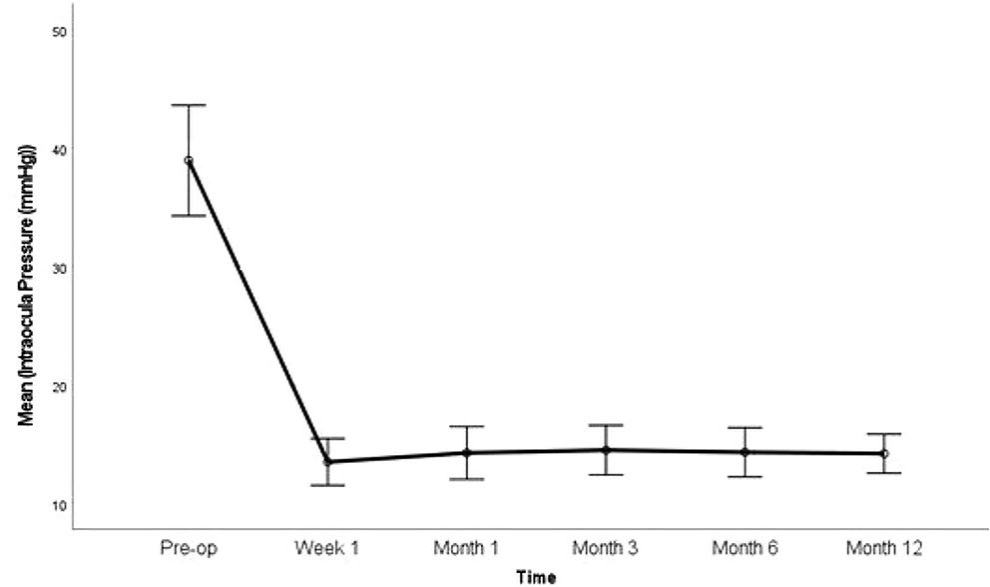
Mean Intraocular Pressure in Preoperative and Postoperative Follow-up Periods (with error bars with 95% Confidence Intervals)

The preoperative and postoperative changes in IOP of the eyes included in the study are shown in Fig. 1; Table 2. While the mean IOP was 38.8 ± 9.2 mmHg preoperatively, it was 13.4 ± 3.9 mmHg at the 1st week, 14.2 ± 4.3 mmHg at the 1st month, 14.6 ± 4.1 mmHg at the 3rd month, 14.3 ± 4.0 mmHg at the 6th month and 16.1 ± 3.3 mmHg at the 12th month post-op. There was a statistically significant difference between postoperative IOP averages in all measurement periods and preoperative IOP averages (*p* < 0.001). The mean decrease between post-op 12th month IOP values and pre-op IOP was 24.76 ± 9.5 (min:11 mmHg decrease, max:40 mmHg decrease) mmHg.

**Table 2:** Intraocular pressures in preoperative and postoperative periods

IOP	Mean ± SD	Median (Min-Max)	*p* ^†^
Pre-op	38.8 ± 9.2	41.0 (24.0–55.0)	
Post-op / Week 1	13.4 ± 3.9	13.0 (8.0–25.0)	< 0.001*
Post-op / Month 1	14.2 ± 4.3	14.0 (8.0–26.0)	< 0.001*
Post-op / Month 3	14.6 ± 4.1	13.0 (8.0–23.0)	< 0.001*
Post-op / Month 6	14.3 ± 4.0	14.0 (9.0–28.0)	< 0.001*
Post-op / Month 12	16.1 ± 3.3	14.0 (11.0–24.0)	< 0.001*

*SD* Standart Deviation, ^*†*^ Wilcoxon Signed Ranks Test, *p < 0.01 (Bonferroni Adjustment)

The change in the mean number of medications used by the patients in the preoperative and postoperative periods is shown in Table 3. While the mean number of medications used preoperatively was 3.6 ± 0.5, it was observed as 1.0 ± 1.1 in the 3rd month, 0.9 ± 1.2 in the 6th month and 0.9 ± 1.2 in the 12th month post-op. The decrease observed in the mean number of medications used after the operation compared to the preoperative period was statistically significant (*p* < 0.001). There was a mean decrease of 2.7 ± 1.3 (min:0, max:4 decrease) in the number of medications used in the post-op 12th month compared to preoperative use.

**Table 3:** Number of medications used in preoperative and postoperative follow-up periods

Number of Anti-Glaucoma Medications	Mean ± SD	Median (Min-Max)	*p* ^†^
Pre-op	3.6 ± 0.5	4.0 (3.0–4.0)	
Post-op / Month 3	1.0 ± 1.1	1.0 (0.0–4.0)	< 0.001*
Post-op / Month 6	0.9 ± 1.2	0.0 (0.0–4.0)	< 0.001*
Post-op / Month 12	0.9 ± 1.2	0.0 (0.0–4.0)	< 0.001*

*SD* Standart Deviation, ^*†*^ Wilcoxon Signed Ranks Test, *p < 0.02 (Bonferroni Adjustment)

**Fig. 2:**
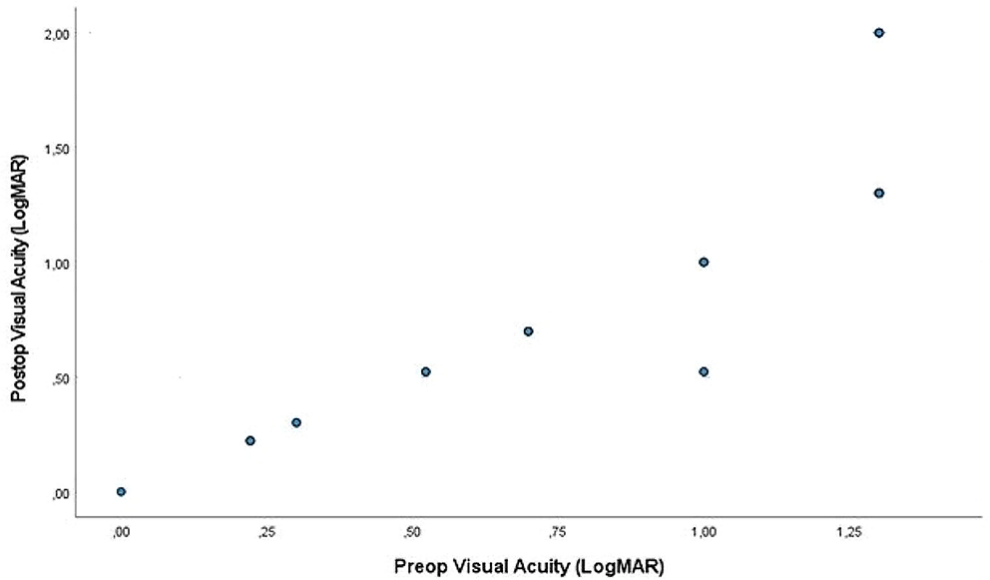
Scatter plot showing best corrected visual acuity changes

Twenty-four eyes had unchanged preoperative and postoperative BCVA (LogMAR) values. BCVA (LogMAR) values of 4 eyes decreased and BCVA (LogMAR) values of 2 eyes increased (Fig. 2). As seen in Table 4, the mean BCVA (LogMAR) values were 0.87 ± 0.46 preoperatively and 0.92 ± 0.60 postoperatively. Preoperative and postoperative visual acuities were similar (*p* > 0.05).

**Table 4:** Preoperative and postoperative best corrected visual acuity (LogMAR)

Visual Acuity (LogMAR)	Mean ± SD	Median (Min-Max)
Pre-operative	0.87 ± 0.46	1.00 (0.00-1.30)
Post-op (at Month 12)	0.92 ± 0.60	1.00 (0.00–2.00)
*p* ^†^ *(Post vs. Pre)*	*0.276*	
Difference (decrease)	0.05 ± 0.27	0.00 ((-0.48)-0.70)

*SD* Standart Deviation, ^*†*^ Wilcoxon Signed Ranks Test

In the early period (3 months), there were four eyes (13.3%) which developed complications due to PGI surgery. Three eyes had choroidal detachment and the other patient had tube exposure. In the long term, no patient developed complications. Throughout the 12-month follow-up period, the studied population notably experienced no occurrences of diplopia, strabismus, or cataract complications.

Only four of the eyes included in the study was unsuccessful because of the need for additional surgical procedure (MP-TDLC) and postoperative IOP did not fall below 21 mmHg. Two of the four eyes had a history of trabeculectomy and MP-TDLC and the other two eyes had a history of trabeculotomy and MP-TDLC; they did not develop complications related to surgery in the early or late period. The number of medications used in the preoperative and postoperative periods (3rd month, 6th month and 12th month) was four. Kaplan-Meier survival analysis showed a success rate of 86.6% during the 12-month follow-up period.

## Discussion

This study investigated the efficacy of a single PGI in managing refractory PCG. The results indicate the cumulative probability of a successful outcome reaching 86.6% within the 12-month follow-up. Within the first three months post-implantation, only four eyes (13.4%) experienced complications; choroidal detachment and tube exposure. Importantly, no further complications were reported in any of the patients during the remainder of the 12-month observation period. These findings underscore the potential of PGI in effectively managing refractory PCG with a low incidence of complications.

In cases where IOP remains uncontrolled following initial surgery, several surgical options are considered. These include filtration surgery with anti-fibrosis drugs, GDDs, or cyclodestructive procedures. GDDs are categorized into open-tube (non-restrictive) and valved (flow-restrictive) devices. Open-tube devices, exemplified by the Molteno and Baerveldt implants, provide non-restricted drainage. In contrast, valved devices, such as the Krupin implant or AGV, regulate flow to minimize complications associated with hypotony during the immediate postoperative period. The Baerveldt and AGV implants are widely used, with smaller sizes available, making them suitable for pediatric cases. Children, in particular, may be prone to implant extrusion and exposure. Non-valved GDDs have been noted to offer sustained and reduced IOP control over an extended period in pediatric patients compared to valved GDDs [14]. In a separate recent study, it was noted that there was no discernible variance in the success rates at 12 and 24 months between eyes implanted with AGV and those with Baerveldt implants within the pediatric population [15]. The AGV, equipped with a flow-restrictive device, has a smaller surface area than the Baerveldt, potentially affecting the extent of IOP reduction and predisposing the patient to a more pronounced hypertensive phase. In a study reported by Ou et al., the cumulative success probability of AGV in patients with PCG was 63% within one year [16]. Razeghinejad et al. report the cumulative success probability of AGV implantation in patients with PCG as 96% ± 4.4% within the first year [10]. In another study comparing AGV and Baerveldt implantation in patients with PCG, the success rates were 53.4% and 78.8%, respectively [17]. There is no study in the literature regarding the success rates of PGI in patients with primary congenital glaucoma. Our study demonstrates that within a one-year follow-up period, the cumulative success probability of PGI reached 86.6%. Complications associated with GDDs include hypotony leading to a shallow anterior chamber and choroidal detachments, tube-cornea touch, tube obstruction, exposed tube or plate, endophthalmitis, and retinal detachment. Careful consideration of implant characteristics and patient factors is crucial in selecting the most appropriate device for optimal outcomes.

In recent times, there has been a notable surge in interest regarding surgical interventions for childhood glaucoma. This is attributed to the adoption of novel surgical techniques used to treat adult glaucoma procedures for congenital glaucoma surgery. Among these innovations, the PGI has emerged as a novel GDD designed to mitigate postoperative complications commonly associated with existing GDDs. Successful applications of the PGI in shunt surgery for glaucoma are reported [13]. The PGI is characterized as a valveless aqueous shunt with specific dimensions, including an endplate surface area of 342.1 mm², a width (leading to trailing edge) of 16.1 mm, and an endplate width (wingspan) of 21.9 mm. Encapsulation around the endplate stands out as one of the predominant causes of failure following GDD surgery. Notably, the endplate surface area of the PGI surpasses that of the AGV, a comparable device. This is believed to contribute to a reduced incidence of encapsulated blebs in the PGI [13]. In our study, the need for bleb revision was not observed in any patient. group. The PGI tube exhibits specific dimensions, with an internal diameter of 0.127 mm, less than half the inner diameter of the AGV. Additionally, the external diameter of the PGI tube is 0.467 mm, representing a significant reduction compared to the AGV. This specific design feature has demonstrated advantageous outcomes for the PGI, as studies consistently show not only adequate reduction in IOP but also a diminished occurrence of hypotony [12, 13]. These attributes underscore the potential clinical benefits of the PGI in the context of glaucoma management, particularly in comparison to the AGV. All subjects included in the study demonstrated buphthalmic eyes. The PGI, known for its pliable, soft silicone construction, was utilized without encountering any size-related complications among the participants. This suggests that the implant adequately conformed to the ocular dimensions of the patients’ eyes. The reduced caliber of the PGI tube represents a significant advantage in mitigating the risk of corneal endothelial damage and lowers the likelihood of conjunctival erosion [12, 18]. Despite its smaller tube diameter and reduced reserve flow capacity, the PGI remains sufficiently sized to offer minimal resistance to aqueous outflow. In our study, IOP was maintained at or below 21 mmHg at the end of the 12-month follow-up period in 26 of 30 eyes (86.6%) which underwent PGI surgery. In addition, the mean number of medications at 12 months was 0.9 ± 1.2.

Postoperative hypotony stands as a significant and potentially vision-threatening complication following GDD surgery in pediatric patients [19]. To address this concern, the PGI tube, being a non-flow restricting implant, incorporates intraluminal stent placement as a preventive measure against hypotony. Instances of hypotonia are reported in patients undergoing PGI surgery with mitomycin C, especially after the removal of Prolene stents [19]. The exact cause of hypotony remains unclear, but it is hypothesized that the use of mitomycin C may lead to excessive filtration, and reduced scarring could contribute to a sudden drop in IOP upon Prolene stent removal. In the present study, mitomycin C was intentionally omitted from the surgical protocol for all PGI procedures. Furthermore, Prolene suture removal occurred uniformly at the one-month mark for all patients, and no instances of hypotonia were observed in this cohort. This approach, abstaining from mitomycin C and timely removal of Prolene stents, was effective in preventing postoperative hypotony in the studied patients. The use of antimetabolites, such as mitomycin C, aims to inhibit fibroblast proliferation and subsequently reduce collagen production, thereby enhancing surgical outcomes. However, this strategy may inadvertently tip the balance toward hyperfiltration, potentially leading to complications like hypotony [7]. The literature on the use of mitomycin C specifically for GDDs in the pediatric cohort is limited and presents conflicting results [20–22].

The occurrence of erosion or extrusion of GDD represents a significant complication uniquely associated with this type of surgery. This complication elevates the risk of endophthalmitis, primarily due to the shunt’s exposure connecting to the anterior chamber. A study notes the incidence of endophthalmitis post-GDD implantation as 9 cases in a sample of 542 eyes [23]. Notably, 6 out of these 9 instances involved tube erosion. In certain situations, even after attempts at repair, the necessity to remove the device arises due to this complication. In contrast, the incidence of extrusion concerning the implant body in patients undergoing PGI surgery is notably lower. A specific surgical series reports only one case of implant body extrusion [24]. Furthermore, in the PGI Study Group series, tube exposure was documented in 4.1% of the patients [12]. This rate of occurrence is higher than that of body extrusion in GDD surgery in general. However, resolving tube exposure is comparatively simpler, typically involving re-covering with tissue such as donor sclera or pericardium [25]. In our study, tube extrusion was observed in a single patient. We hypothesize that the inadequacy of tube stabilization through suturing contributed to this occurrence. To address this, we reinforced the stabilization of the tube in the affected patient and subsequently re-covered it using pericardium, sealing it finally with a conjunctival autograft.

The present study is constrained by its small sample size, retrospective nature, short follow-up period and lack of a control group. Preoperative and postoperative corneal endothelial density assessments were not conducted in our study. Future investigations should involve larger cohorts and extend follow-up durations to comprehensively assess the efficacy and safety of PGI in refractory PCG patients.

To the best of our knowledge, this study is the first to describe the safety and efficacy of PGI in the treatment of patients with refractory PCG. The findings of this investigation reveal that PGI possesses a notably high rate of both complete and qualified success. Additionally, there was a significant reduction in IOP and a decrease in the number of medications post-implantation. Moreover, the PGI is characterized by an advantageous safety profile, complemented by minimal post-operative care requirements. The outcomes of this study are promising, highlighting the potential of PGI as a viable therapeutic option in the realm of glaucoma treatment. There is a need for comparative studies that evaluate PGI against other glaucoma surgical procedures and different types of GDD.
